# Association between BDNF methylation and expression of the NLRP3 inflammasome in the brain is disrupted in Alzheimer’s disease

**DOI:** 10.1007/s00702-026-03280-3

**Published:** 2026-10-03

**Authors:** Hao Tang, Helene A. Fachim, Michael K. Harte, Gavin P. Reynolds

**Affiliations:** 1 Division of Biosciences Education, School of Biological and Biomedical Sciences, College of Life Sciences, University of Leicester, Leicester, UK; 2 Department of Diabetes and Endocrinology, Salford Royal Hospital, Salford, UK; 3 Division of Pharmacy and Optometry, School of Health Sciences, Faculty of Biology, Medicine and Health, University of Manchester, Manchester, UK; 4 Biomolecular Sciences Research Centre, Sheffield Hallam University, Howard Street, Sheffield, S1 0WB UK

**Keywords:** Alzheimer’s disease, DNA methylation, BDNF, Neuroinflammation, NLRP3 inflammasome

## Abstract

Activation of the NLRP3 inflammasome pathway and dysfunction of brain-derived neurotrophic factor (BDNF) are both implicated in the pathogenesis of Alzheimer’s disease (AD). While in vitro and in vivo models support these mechanisms, there is little research investigating their relationship in human brain tissue, and whether this relationship may be abnormal in the AD brain. Temporal cortex tissue taken post-mortem from AD patients and control subjects was analysed for expression of genes for the NLRP3 pathway (NLRP3, PYCARD, CASP1), the potassium channel THIK1 (KCNK13), and related pro-inflammatory cytokines (IL-1β, IL-18). BDNF mRNA expression and DNA methylation in the BDNF exon IV promoter were determined. Correlations between these BDNF measures and NLRP3-related genes were investigated. A significant relationship between BDNF mRNA expression and mean methylation was observed in control subjects but not in AD. In controls, significant correlations with both BDNF expression and mean BDNF methylation were observed for NLRP3, CASP1 and IL1B. These correlations were lost in the AD group, which also showed a significant correlation of KCNK13 expression with both BDNF measures, not seen in controls. This study indicates that the relationship between BDNF and NLRP3 activation, as measured by NLRP3 inflammasome components and proinflammatory cytokine products, is disrupted in AD. The contrary correlation emerging in AD between BDNF measures and KCNK13 expression, shown to be elevated in AD, indicates the possible relationship of this finding with BDNF dysfunction. These findings enhance our understanding of AD pathophysiology, demonstrating the importance of understanding the neuroinflammatory-neurotrophic balance in neurodegenerative processes.

## Introduction

Alzheimer’s disease (AD) is a neurodegenerative disorder and the major cause of dementia in the aging population (Chowdhary et al. 2022). Amyloid beta (Aβ) aggregates and intracellular deposition of neurofibrillary tangles containing phosphorylated tau protein are the characteristic pathological hallmarks of the disease. However, the relationship of this proteinopathy to, and the mechanisms involved in, the neurodegenerative process which results in profound neuronal damage and death remains unclear. Several factors contributing to this neurodegeneration, in addition to amyloid cascade and tau hyperphosphorylation, include neuroinflammation, oxidative stress and failure of neurotransmitter systems (Dash et al. 2025; Nimgampalle et al. 2023; Zhou et al. 2022). These known pathologies, as well as currently undetected ones, likely interact with each other, complicating the understanding of the mechanisms underlying neurodegeneration and challenging the development of treatment.

One inflammatory mechanism potentially important in AD is an activation of the NOD-like receptor pyrin domain containing 3 inflammasome (NLRP3). This comprises a complex of three proteins: the cytosolic sensor molecule NLRP3, an apoptosis-associated speck-like protein containing a caspase-activating recruitment domain (ASC, coded by the PYCARD gene) and an effector molecule, cysteine protease pro-caspase-1 (CASP1) (Rathinam and Fitzgerald 2016). The assembly of ASC with NLRP3 provides the platform for activating pro-caspase-1, leading to caspase-1 recruitment, oligomerization and subsequent pro-enzyme activation (Kelley et al. 2019). Cleavage of pro-interleukins by caspase-1 can produce the mature, biologically activated forms of IL-1β and IL-18 (Lamkanfi and Dixit 2014). While in vitro and in vivo model systems provide strong support for NLRP3 involvement (Heneka et al. 2013; Ising et al. 2019), we previously reported no significant differences between AD and control brain tissue in the gene expression of components of the NLRP3 pathway (Tang and Harte 2021). Nevertheless, a dysfunction leading to increased inflammation, that may be mediated by the NLRP3 inflammasome, remains a potential mechanism contributing to the neurodegenerative process in AD.

Brain-derived neurotrophic factor (BDNF) is also implicated in AD. BDNF is involved in the survival, development, and function of neurons as well as being a critical factor in synaptic plasticity; it is therefore important in learning and memory processes and their dysfunction (Azman and Zakaria 2022). As such it has been studied in several neurological and psychiatric diseases, including AD. BDNF depletion is associated with several of the pathological hallmarks of AD, notably tau phosphorylation and Aβ accumulation, as well as neuroinflammation and neuronal apoptosis (Numakawa and Odaka 2021). Aberrations in BDNF expression and its mechanisms of regulation have therefore attracted significant attention due to its potential role in the pathophysiology of the disease.

DNA methylation is an epigenetic mechanism that involves the addition of methyl groups to the DNA molecule, often resulting in gene silencing. In the context of AD, changes in methylation of the BDNF gene have been observed; increased methylation of BDNF promoter regions is associated with reduced gene expression in AD, both in the brain (Rao et al. 2012) and peripheral blood (Nagata et al. 2015; Xie et al. 2017). Notwithstanding some negative reports (Fransquet et al. 2020; Aarons et al. 2019), this hypermethylation has been proposed as a mechanism contributing to a downregulation of BDNF, exacerbating neuronal dysfunction and cognitive deficits in AD.

The mechanisms underlying an altered DNA methylation of BDNF in AD are not entirely understood but may involve oxidative stress, inflammation, and dysregulation of enzymes involved in DNA methylation, such as the DNA methyltransferases (Cheng et al. 2024). Chronic inflammation, a hallmark of AD, can influence epigenetic modifications, leading to changes in gene expression that affect neuronal health and function. Understanding the relationship between BDNF expression, DNA methylation, neuroinflammation and AD is therefore valuable in opening avenues for potential therapeutic interventions.

**Fig. 1:**
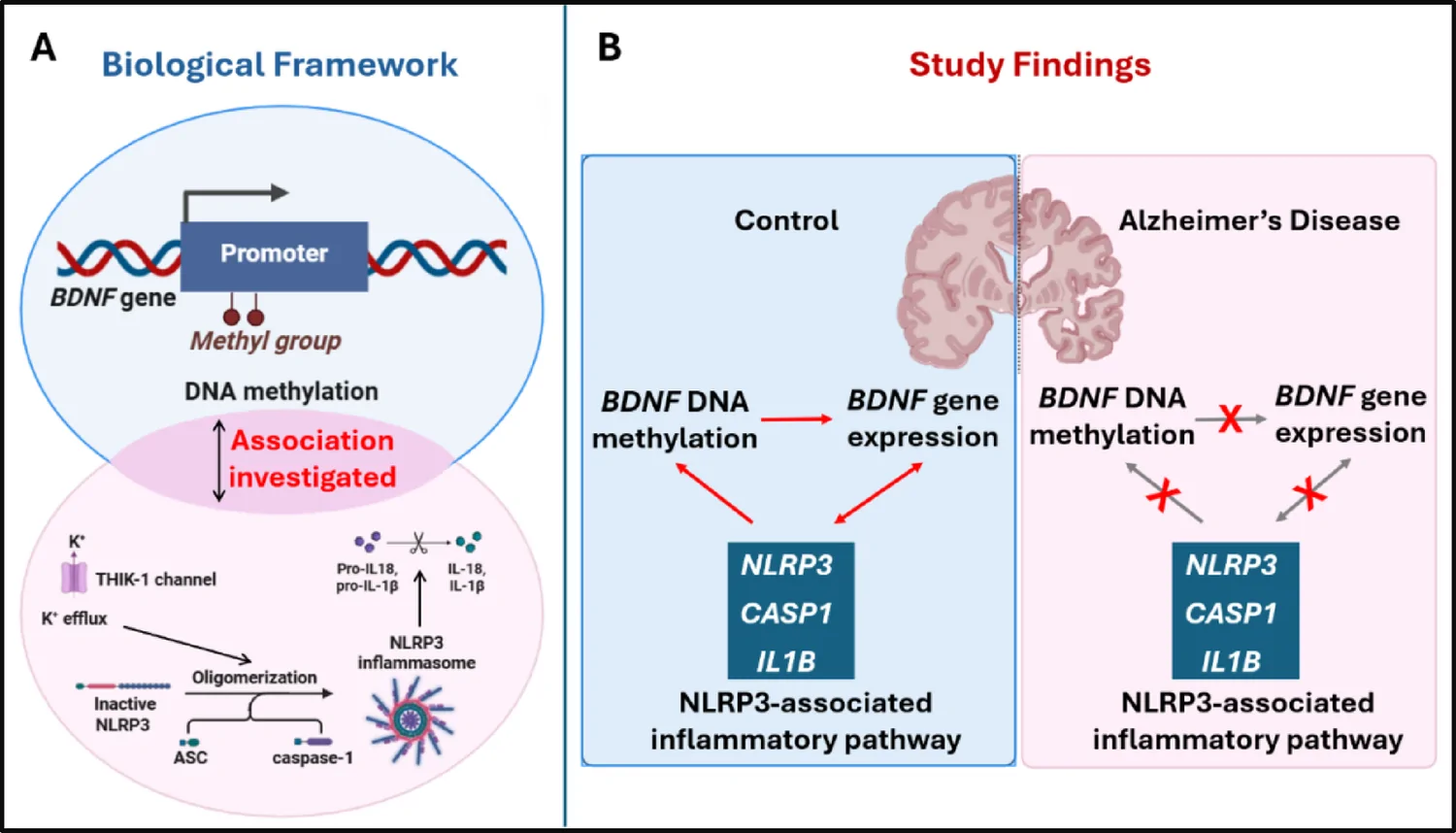
Relationship between BDNF regulation and NLRP3-associated inflammatory signalling in control and Alzheimer’s disease temporal cortex. **A** Schematic biological framework illustrating BDNF regulation and NLRP3-associated inflammatory signalling as two related molecular systems; the overlapping region indicates the association investigated in the present study and does not imply a direct physical or cellular interaction. **B** Summary of the study findings in control (left) and AD (right) temporal cortex. In controls, BDNF mRNA expression and mean BDNF exon IV methylation are significantly correlated with NLRP3, CASP1 and IL1B, while these BDNF–inflammatory signalling relationships are disrupted in AD. Image created using BioRender

It is established that inflammation can have effects on BDNF function such that an inflammatory challenge can result in reduced BDNF with consequences that may include reductions in the functional integrity of neurons (Golia et al. 2019). This interaction between inflammation and BDNF extends to mechanisms involving the NLRP3 inflammasome (Fu et al. 2020) (Fig. 1A). In the current study we have investigated the expression and DNA methylation of a regulatory sequence in BDNF in post mortem brain tissue in AD and control subjects and its relationship to the expression of components of the NLRP3 pathway (Fig. 1B). These include the genes NLRP3, PYCARD and CASP1, KCNK13 – coding for the upstream regulatory potassium channel THIK1 which we have previously shown to be increased in expression in AD (Tang et al. 2023) – and IL1B and IL18, genes for the downstream proinflammatory cytokines activated by caspase-1.

## Methods

Post mortem AD brain tissue from the temporal cortex was obtained from the Manchester Brain Bank at Salford Royal NHS Foundation Trust. Matched control samples were also supplied by the brain bank. The Manchester Brain Bank has been approved by the Newcastle & North Tyneside 1 Research Ethics Committee (REC reference 09/H0906/52 + 5). Under conditions agreed with the Research Ethics Committee, tissue or data can be supplied for research without the requirement for individual researchers to apply for further ethical approval. All tissue collection, storage and use followed the requirements of the UK Human Tissue Act. All samples were anonymised and taken from brains that had undergone neuropathological assessment. AD cases had a clinical diagnosis of dementia and were all defined as having moderate or advanced Alzheimer’s disease on the basis of Braak staging for the extent of neurofibrillary tangles (stages III-IV and V-VI respectively). The majority of cases also had assessment of amyloid plaques; these results were fully consistent with other clinical and neuropathological diagnostic criteria.

Standard procedures were employed with tissue storage, handling and examination according to UK research ethics. Unfixed samples were stored at − 80^◦^C immediately after dissection until tissue homogenization. Research was conducted in compliance with the principles of the Declaration of Helsinki. Demographic parameters and clinical diagnosis were provided by the brain bank.

### Quantitative real-time PCR

RNA expression was quantified using reverse transcriptase semi-quantitative real-time PCR (RT-qPCR) as previously described (Tang and Harte 2021). All the procedures were performed according to the MIQE guidelines (Bustin et al. 2009). Briefly, total RNA was extracted using TRIzol™ Reagent (Thermo Fisher, UK) followed by RNA clean up using Monarch^®^ RNA Cleanup Kit (New England Biolabs, UK). RNA purity and concentrations were assessed using a NanoDrop 2100 (Thermo Fisher, UK). Single-stranded cDNA was synthesized from 1 ng of total RNA using M-MLV Reverse Transcriptase (ThermoFisher, UK). cDNA concentrations were measured on a NanoDrop 2100 and diluted to 500 ng/µL in RNase-free water.

Reference genes (RGs) were selected as previously described (Tang and Harte 2021). Primer pairs were designed, synthesized and specificity analysed by dissociation curve. RT-qPCR reactions were carried out using Power SYBR™ Green PCR Master Mix (ThermoFisher, UK) on a 7900HT Real-Time PCR System with 384-well format (Applied Biosystems, UK). The final volume for each reaction was 10 µL with 1 µL (0.25 µM) of corresponding primers, 1 µL (500 ng/µL) of total cDNA, 3 µL RNase-free water, and 5 µL SYBR green. All samples were run in triplicate. The thermal cycler parameters were as follows: UDG activation at 50^◦^C for 2 min, and DNA polymerase activation at 95^◦^C for 10 min, followed by amplification of cDNA for 40 cycles with denaturation at 95^◦^C for 15 s and annealing/extension at respective annealing temperature for each gene for 1 min. Dissociation curve analyses were carried out at the end of each run for PCR product verification. The intra-assay variation was controlled as standard deviation of triplicates < 0.2 and was analysed by using raw Ct value of RGs and there were no significant variations between plates.

### DNA extraction, bisulphite conversion, and pyrosequencing

Genomic DNA from temporal cortex of human postmortem tissue of AD and matched controls was purified using QIAamp DNA Mini Kit (Qiagen) according to the manufacturer’s instructions. EpiTec Fast DNA Bisulfite Kit (Qiagen) was used to convert unmethylated cytosine to uracil in the CpG sites with a calculated mean conversion of 99%. For analysis, a previously-studied sequence in the BDNF exon IV promoter containing transcription factor binding sites was amplified by PCR, the percentage methylation at each of four CpG sites determined with a PyroMark Q48 Autoprep (Qiagen UK). PCR reactions, amplification conditions and preparation of forward, reverse and sequencing primers were carried out according to our previous studies (Fachim et al. 2021).

Statistical analysis was undertaken using SPSS v30. Spearman rank tests were used to identify significant correlations, and t-tests or Z-tests were used to identify differences between groups or correlations respectively.

## Results

A series of 78 AD and 37 control tissue samples were studied, with details listed in Table 1. Occasional reduced numbers, as shown in the following tables, reflected occasional failures in PCR amplification or pyrosequencing. The AD and control groups were matched closely for age, sex and post-mortem delay.

**Fig. 2:**
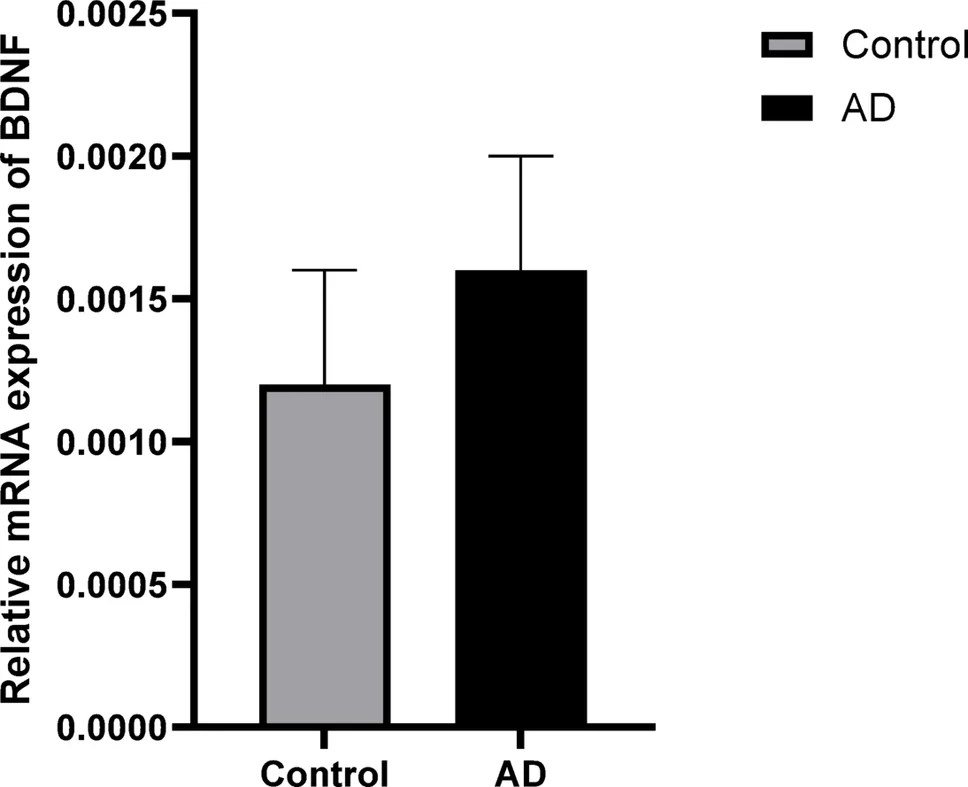
Relative mRNA expression of BDNF in temporal cortex in control and Alzheimer’s disease. Mean BDNF mRNA levels were higher in the AD group compared to controls; however, this difference did not reach statistical significance (*p* = 0.36). Data shown as mean ± SEM

Mean mRNA expression of BDNF was increased in the AD sample above controls but did not reach statistical significance (*p* = 0.36; Fig. 2). DNA methylation of BDNF also showed no significant difference (Table 2), although a non-significant reduction, particularly at the CpG2 site which includes the CREB transcription factor recognition site (Kundakovic et al. 2015), was apparent in the AD group.

**Table 1:** Demographic and clinical characteristics of study subjects

	Control (*n* = 37)	AD (*n* = 78)
Age of death (Years)	82.6 ± 11.5	81.5 ± 8.8
Sex	M = 19, F = 18	M = 41, F = 37
Post-Mortem Interval (Hours)	81.2 ± 36.9	83.2 ± 40.9

**Table 2:** BDNF gene methylation

	Control	AD	Diff. between groups
BDNF methylation (%)	(*n* = 33)	(*n* = 63)	t (p)
CpG1	5.1 ± 1.8	5.2 ± 1.9	0.15 (0.880)
CpG2	5.2 ± 2.8	4.3 ± 2.1	1.67 (0.099)
CpG3	5.3 ± 2.1	4.8 ± 2.2	1.14 (0.257)
CpG4	9.0 ± 3.6	8.9 ± 2.5	0.07 (0.947)
Mean	6.2 ± 1.8	5.8 ± 1.5	1.02 (0.312)
Correlation of gene expression with mean methylation	0.383 (*p* = 0.030)	−0.169 (*p* = 0.185)	Z = 2.54, *p* = 0.011

Data expressed as mean ± standard deviation

**Table 3:** Correlations of BDNF expression with expression of genes of the NLRP3 pathway

Gene	Control (*n* = 36–37)	AD (*n* = 72–78)	Diff between groups
NLRP3	**0.470 (0.003)**	0.109 (0.355)	ns
CASP1	**0.639 (0.001)**	**−0.365 (0.001)**	Z = 5.50 (0.000)
PYCARD	−0.081 (0.634)	−0.091 (0.430)	ns
IL1B	**−0.357 (0.030)**	−0.142 (0.215)	ns
IL18	−0.062 (0.716)	−0.069 (0.549)	ns
KCNK13	−0.316 (0.057)	**0.563 (0.000)**	Z = 4.65 (0.000)

Data are Spearman rank correlation coefficients with p values in brackets. Significant correlations are shown in bold

**Table 4:** Correlations of BDNF methylation with expression of genes of the NLRP3 pathway

Gene	Control (*n* = 31–32)	AD (*n* = 59–63)	Diff between groups
NLRP3	**−0.596 (0.000)**	−0.063 (0.633)	Z = 2.76 (0.006)
CASP1	**−0.411 (0.020)**	−0.071 (0.588)	ns
PYCARD	−0.175 (0.338)	0.174 (0.174)	ns
IL1B	**0.481 (0.005)**	0.206 (0.105)	ns
IL18	**0.456 (0.009)**	−0.033 (0.796)	Z = 2.03 (0.042)
KCNK13	0.074 (0.686)	**−0.260 (0.041)**	ns

Data are Spearman rank correlation coefficients with p values in brackets. Significant correlations are shown in bold

Mean methylation was significantly correlated with BDNF expression in control subjects (Spearman’s rho = 0.383; *p* = 0.030), primarily reflecting an effect at the CpG2 site, but this was not found in the AD group (rho=−169; *p* = 0.185). The correlation coefficients of the two groups differed significantly (Z = 2.54; *p* = 0.011).

Table 3 demonstrates the correlations between expression of several NLRP3-related genes and that of BDNF. In control subjects, mRNA expression of BDNF was positively correlated with NLRP3 and CASP1, and negatively with IL1beta in the control series. The correlations with NLRP3, CASP1, and IL1B were all lost in the AD group, with CASP1 showing a negative correlation and a significant difference in correlation between the groups. KCNK13, showing a non-significant negative correlation with BDNF expression in controls, demonstrated a significantly different and positive correlation in the AD group.

Correlations of BDNF mean methylation with expression of NLRP3-related genes are shown in Table 4. Significant correlations with NLRP3, CASP1, IL1B and IL18 in the control group are all lost in AD tissue, an effect that demonstrates significant differences between groups for NLRP3 and IL18. A significant correlation of KCNK13 with BDNF methylation emerges only in the AD group. We also undertook an exploratory regression analysis of the effect of expression of the NLRP3-related genes on BDNF methylation in each group. A highly significant relationship was observed in the controls (F = 5.960, *p* < 0.001) which was lost in AD (F = 1.172, *p* = 0.138).

## Discussion

These findings demonstrate a relationship between correlates of BDNF transcription – promoter DNA methylation and gene expression – and the expression of NLRP3-related genes in the human brain. These relationships, observed in post-mortem human temporal cortical brain tissue from individuals with age-consistent normal neuropathology, appear to be lost in tissue samples from AD subjects. They are seen in the absence of any significant changes in either these BDNF measures or NLRP3 gene expression in AD. One exception to this is apparent; expression of the THIK1 gene KCNK13, which has a tendency to an inverse relationship with BDNF expression in control subjects, shows a strong and positive correlation in AD tissue, the latter also reflected in the association with BDNF methylation. While we primarily employed rank correlation analyses as a conservative approach avoiding the assumptions in parametric analyses, undertaking a linear regression analysis confirmed our findings in which a highly significant relationship between expression of the six NLRP3 genes and BDNF methylation was observed in control samples but lost in AD.

Some studies have identified a marked reduction in BDNF protein (Hock et al. 2000) and mRNA expression (Holsinger et al. 2000) in the brains and peripheral blood (Gezen-Ak et al. 2013) of individuals with AD, in which the reported reduction correlates with cognitive decline and the neuronal loss characteristic of AD. However while reductions in BDNF are frequently reported, reviews report inconsistencies (Du et al. 2018; Cade et al. 2022) with conflicting findings including increases in the brain (Durany et al. 2000) and increased blood BDNF concentrations in AD patients (Faria et al. 2014). While one interpretation is that such increases may reflect a compensatory activation and/or release of BDNF (Faria et al. 2014), measurement of BDNF in plasma and serum is liable to many artefactual influences that may produce misleading findings (Gejl et al. 2019). Furthermore, there are multiple peripheral sources of BDNF which indicate that blood concentrations and their changes are not solely associated with CNS function. Previous studies also suggest the possible role of neurotrophic factors in addition to BDNF in the pathology of AD (Du et al. 2018; Durany et al. 2000).

We have previously reported that expression of NLRP3 components in the AD temporal cortex is not significantly different from control subjects (Tang and Harte 2021). However, we did observe an elevation in glial fibrillary acidic protein, a marker of astrocytic activation. We have also identified an elevation of KCNK13 expression and a corresponding reduction in KCNK13 promoter methylation in the AD brain, again using temporal cortex tissue samples that have been studied here (Tang et al. 2023).

There is a significant positive correlation between BDNF gene expression and BDNF methylation, an unexpected finding since increased methylation is considered to suppress transcription. However, it is notable that we find lower BDNF methylation, but increased BDNF expression, are both associated with increased expression of NLRP3 and CASP1, and a reduced expression of IL1B. While life events may influence BDNF methylation (Fachim et al. 2021), it does appear that changes in inflammatory status can occur in parallel with BDNF methylation (e.g. Al-Zharani et al. 2025) suggesting a possibe influence of inflammation on BDNF methylation.

However, whatever underlies them, these “normal” relationships between BDNF and inflammatory factors, seen in age-matched control brain tissue which is relatively unaffected by neurodegenerative processes, are lost in AD brain. The effect is apparent for correlations with both expression and BDNF methylation, indicating a disruption of the normal, presumably homeostatic, relationships between these measures of inflammatory activity and the protective neurotrophic factor of BDNF.

It is notable how the relationships with KCNK13 expression are the opposite of those found for NLRP3 and its related genes. We previously showed KCNK13 expression to be activated in the AD brain tissue as well as in an animal model of AD. That this ion-channel gene, coding for THIK1 which contributes to the control of NLRP3 inflammasome function, is upregulated in the AD brain is interesting and our findings suggest an involvement, absent in the control subjects, of BDNF in this process – although whether causal or consequential is unclear.

There are many limitations to consider in this preliminary study. Measurement of gene expression in the post-mortem brain is compromised by the degeneration of RNA; this may not always be accurately compensated for by concurrent measurement of housekeeping gene expression. This is one reason we chose to investigate DNA methylation for BDNF, which is a relatively stable measure and potential correlate of gene expression. However none of the reported measures showed a significant correlation with the post-mortem interval. Further studies investigating protein products would be a valuable approach to corroborating these findings. We were limited to the study of one brain region strongly affected by changes associated with AD; future work including the study of other regions, particularly the hippocampus, would be more informative. Our sample size is substantial for a post mortem study, and brains had undergone neuropathological assessment, although factors such as cause of death and agonal state have not been accounted for and may influence measurements, particularly for RNA.

There remains much to understand about these findings. As yet it is unclear what the direction of causality is: whether, in the normal brain, the control of BDNF expression is changing in response to inflammasome activation, or vice versa. Certainly there is evidence for both processes and it is likely there is a dynamic interrelationship between BDNF expression and inflammatory changes in the brain (Al-Zharani et al., 2025; Dadkhah et al. 2024).

Notwithstanding the preliminary nature of this study, and the various unanswered questions that it raises, the relationships seen in control brains provide evidence for an interrelationship between BDNF function and one aspect of neuroinflammatory activity associated with the innate immune response in the human brain. The loss of this relationship in AD identifies a new feature of the disruption of neural function in the disease; understanding what specific factors underlie this could provide novel opportunities for intervention in ameliorating the consequences of the chronic inflammatory processes occurring in AD.

## Data Availability

Original data are available from the corresponding author upon reasonable request.

## References

[CR1] Aarons T, Bradburn S, Robinson A, Payton A, Pendleton N, Murgatroyd C (2019) Dysregulation of BDNF in prefrontal cortex in Alzheimer’s disease. J Alzheimers Dis 69(4):1089–1097. 10.3233/JAD-190049 31127785

[CR2] Al-Zahrani NS, Zafrah H, Hassan AH, El Nashar EM, El Henafy HMA (2025) Zinc oxide nanoparticles exposure disrupts brain redox-inflammatory-epigenetic axis and impairs PI3K/Akt survival pathway in male offspring. Metab Brain Dis 40(7):257. 10.1007/s11011-025-01699-3 40892347

[CR3] Azman KF, Zakaria R (2022) Recent advances on the role of brain-derived neurotrophic factor (BDNF) in neurodegenerative diseases. Int J Mol Sci 23(12):6827. 10.3390/ijms23126827 PMC9224343 35743271

[CR4] Bustin SA, Benes V, Garson JA, Hellemans J, Huggett J, Kubista M, Mueller R, Nolan T, Pfaffl MW, Shipley GL, Vandesompele J, Wittwer CT (2009) The MIQE guidelines: minimum information for publication of quantitative real-time PCR experiments. Clin Chem 55(4):611–622. 10.1373/clinchem.2008.112797 19246619

[CR5] Cade S, Zhou XF, Bobrovskaya L (2022) The role of brain-derived neurotrophic factor and the neurotrophin receptor p75NTR in age-related brain atrophy and the transition to Alzheimer’s disease. Rev Neurosci 33(5):515–529. 10.1515/revneuro-2021-0111 34982865

[CR6] Cheng R, Shu J, Chen H, Li M, Cheng X, Liu L (2024) DNA Methylation in Aging and Alzheimer’s Disease. Hum brain (Hong Kong) 3(3). 10.37819/hb. 37813.32027 10.37819/hb.3.2027 PMC12178119 40538761

[CR7] Chowdhary N, Barbui C, Anstey KJ, Kivipelto M, Barbera M, Peters R, Zheng L, Kulmala J, Stephen R, Ferri CP (2022) Reducing the risk of cognitive decline and dementia: WHO recommendations. Front Neurol 12:765584. 10.3389/fneur.2021.765584 PMC8784726 35082745

[CR8] Dadkhah M, Baziar M, Rezaei N (2024) The regulatory role of BDNF in neuroimmune axis function and neuroinflammation induced by chronic stress: a new therapeutic strategies for neurodegenerative disorders. Cytokine 174:156477. 10.1016/j.cyto.2023.156477 38147741

[CR9] Dash UC, Bhol NK, Swain SK, Samal RR, Nayak PK, Raina V, Panda SK, Kerry RG, Duttaroy AK, Jena AB (2025) Oxidative stress and inflammation in the pathogenesis of neurological disorders: Mechanisms and implications. Acta Pharm Sinica B 15(1):15–34. 10.1016/j.apsb.2024.10.004 PMC11873663 40041912

[CR10] Du Y, Wu HT, Qin XY, Cao C, Liu Y, Cao ZZ, Cheng Y (2018) Postmortem brain, cerebrospinal fluid, and blood neurotrophic factor levels in Alzheimer’s disease: a systematic review and meta-analysis. J Mol Neurosci 65(3):289–300. 10.1007/s12031-018-1100-8 29956088

[CR11] Durany N, Michel T, Kurt J, Cruz-Sánchez FF, Cervós‐Navarro J, Riederer P (2000) Brain‐derived neurotrophic factor and neurotrophin‐3 levels in Alzheimer’s disease brains. Int J Dev Neurosci 18(8):807–13. 10.1016/S0736-5748(00)00046-0 11154850

[CR12] Fachim HA, Corsi-Zuelli F, Loureiro CM, Iamjan S-a, Shuhama R, Joca S, Menezes PR, Heald A, Louzada-Junior P, Dalton CF (2021) Early-life stress effects on BDNF DNA methylation in first-episode psychosis and in rats reared in isolation. Prog Neuropsychopharmacol Biol Psychiatry 108:110188. 10.1016/j.pnpbp.2020.110188 33259836

[CR13] Faria MC, Gonçalves GS, Rocha NP, Moraes EN, Bicalho MA, Cintra MTG, de Paula JJ, de Miranda LFJR, de Souza Ferreira AC, Teixeira AL (2014) Increased plasma levels of BDNF and inflammatory markers in Alzheimer’s disease. J Psychiatr Res 53:166–172. 10.1016/j.jpsychires.2014.01.019 24576746

[CR14] Fransquet PD, Ritchie K, Januar V, Saffery R, Ancelin M-L, Ryan J (2020) Is peripheral BDNF promoter methylation a preclinical biomarker of dementia? J Alzheimer’s Disease 73(2):645–655. 10.3233/JAD-190738 31839607

[CR15] Fu Q, Li J, Qiu L, Ruan J, Mao M, Li S, Mao Q (2020) Inhibiting NLRP3 inflammasome with MCC950 ameliorates perioperative neurocognitive disorders, suppressing neuroinflammation in the hippocampus in aged mice. Int Immunopharmacol 82:106317. 10.1016/j.intimp.2020.106317 32087497

[CR16] Gejl AK, Enevold C, Bugge A, Andersen MS, Nielsen CH, Andersen LB (2019) Associations between serum and plasma brain-derived neurotrophic factor and influence of storage time and centrifugation strategy. Sci Rep 9(1):9655. 10.1038/s41598-019-45976-5 PMC6609657 31273250

[CR17] Gezen-Ak D, Dursun E, Hanağası H, Bilgiç B, Lohman E, Araz ÖS, Atasoy İL, Alaylıoğlu M, Önal B, Gürvit H (2013) BDNF, TNFα, HSP90, CFH, and IL-10 serum levels in patients with early or late onset Alzheimer’s disease or mild cognitive impairment. J Alzheimers Dis 37(1):185–195. 10.3233/JAD-130497 23948885

[CR18] Golia MT, Poggini S, Alboni S, Garofalo S, Albanese NC, Viglione A, Ajmone-Cat MA, St-Pierre A, Brunello N, Limatola C (2019) Interplay between inflammation and neural plasticity: both immune activation and suppression impair LTP and BDNF expression. Brain Behav Immun 81:484–494. 10.1016/j.bbi.2019.07.003 31279682

[CR19] Heneka MT, Kummer MP, Stutz A, Delekate A, Schwartz S, Vieira-Saecker A, Griep A, Axt D, Remus A, Tzeng T-C (2013) NLRP3 is activated in Alzheimer’s disease and contributes to pathology in APP/PS1 mice. Nature 493(7434):674–678. 10.1038/nature11729 PMC3812809 23254930

[CR20] Hock C, Heese K, Hulette C, Rosenberg C, Otten U (2000) Region-specific neurotrophin imbalances in Alzheimer disease: decreased levels of brain-derived neurotrophic factor and increased levels of nerve growth factor in hippocampus and cortical areas. Arch Neurol 57(6):846–851. 10.1001/archneur.57.6.846 10867782

[CR21] Holsinger RD, Schnarr J, Henry P, Castelo VT, Fahnestock M (2000) Quantitation of BDNF mRNA in human parietal cortex by competitive reverse transcription-polymerase chain reaction: decreased levels in Alzheimer’s disease. Mol Brain Res 76(2):347–354. 10.1016/S0169-328X(00)00023-1 10762711

[CR22] Ising C, Venegas C, Zhang S, Scheiblich H, Schmidt SV, Vieira-Saecker A, Schwartz S, Albasset S, McManus RM, Tejera D (2019) NLRP3 inflammasome activation drives tau pathology. Nature 575(7784):669–673. 10.1038/s41586-019-1769-z PMC7324015 31748742

[CR23] Kelley N, Jeltema D, Duan Y, He Y (2019) The NLRP3 inflammasome: an overview of mechanisms of activation and regulation. Int J Mol Sci 20(13):3328. 10.3390/ijms20133328 PMC6651423 31284572

[CR24] Kundakovic M, Gudsnuk K, Herbstman JB, Tang D, Perera FP, Champagne FA (2015) DNA methylation of BDNF as a biomarker of early-life adversity. Proc Natl Acad Sci U S A 112(22):6807–6813. 10.1073/pnas.1408355111 PMC4460453 25385582

[CR25] Lamkanfi M, Dixit VM (2014) Mechanisms and functions of inflammasomes. Cell 157(5):1013–1022. 10.1016/j.cell.2014.04.007 24855941

[CR26] Nagata T, Kobayashi N, Ishii J, Shinagawa S, Nakayama R, Shibata N, Kuerban B, Ohnuma T, Kondo K, Arai H (2015) Association between DNA methylation of the BDNF promoter region and clinical presentation in Alzheimer’s disease. Dement geriatric Cogn disorders extra 5(1):64–73. 10.1159/000375367 PMC4376924 25873928

[CR27] Nimgampalle M, Chakravarthy H, Sharma S, Shree S, Bhat AR, Pradeepkiran JA, Devanathan V (2023) Neurotransmitter systems in the etiology of major neurological disorders: emerging insights and therapeutic implications. Ageing Res Rev 89:101994. 10.1016/j.arr.2023.101994 37385351

[CR28] Numakawa T, Odaka H (2021) Brain-derived neurotrophic factor signaling in the pathophysiology of Alzheimer’s disease: beneficial effects of flavonoids for neuroprotection. Int J Mol Sci 22(11):5719. 10.3390/ijms22115719 PMC8199014 34071978

[CR29] Rao J, Keleshian V, Klein S, Rapoport S (2012) Epigenetic modifications in frontal cortex from Alzheimer’s disease and bipolar disorder patients. Translational psychiatry 2(7):e132–e132. 10.1038/tp.2012.55 PMC3410632 22760556

[CR30] Rathinam VA, Fitzgerald KA (2016) Inflammasome complexes: emerging mechanisms and effector functions. Cell 165(4):792–800. 10.1016/j.cell.2016.03.046 PMC5503689 27153493

[CR31] Tang H, Harte M (2021) Investigating markers of the NLRP3 inflammasome pathway in Alzheimer’s disease: a human post-mortem study. Genes (Basel) 12(11):1753. 10.3390/genes12111753 PMC8622528 34828359

[CR32] Tang H, Sun Y, Fachim HA, Cheung TKD, Reynolds GP, Harte MK (2023) Elevated expression of two pore potassium channel THIK-1 in Alzheimer’s disease: an inflammatory mechanism. J Alzheimer’s Disease 95(4):1757–1769. 10.3233/JAD-230616 37718820

[CR33] Xie B, Xu Y, Liu Z, Liu W, Jiang L, Zhang R, Cui D, Zhang Q, Xu S (2017) Elevation of peripheral BDNF promoter methylation predicts conversion from amnestic mild cognitive impairment to Alzheimer’s disease: a 5-year longitudinal study. J Alzheimers Dis 56(1):391–401. 10.3233/JAD-160954 27935556

[CR34] Zhou X, Venigalla M, Raju R, Münch G (2022) Pharmacological considerations for treating neuroinflammation with curcumin in Alzheimer’s disease. J Neural Transm 129(5):755–771. 10.1007/s00702-022-02480-x 35294663

